# A dual mode electronic synapse based on layered SnSe films fabricated by pulsed laser deposition†

**DOI:** 10.1039/c9na00447e

**Published:** 2020-01-20

**Authors:** Xinxin Chen, Chun-Hung Suen, Hei-Man Yau, Feichi Zhou, Yang Chai, Xiaodan Tang, Xiaoyuan Zhou, Nicolas Onofrio, Ji-Yan Dai

**Affiliations:** Department of Applied Physics, The Hong Kong Polytechnic University Hung Hom Kowloon Hong Kong jiyan.dai@polyu.edu.hk; College of Physics, Chongqing University Chongqing 401331 P. R. China

## Abstract

An artificial synapse, such as a memristive electronic synapse, has caught world-wide attention due to its potential in neuromorphic computing, which may tremendously reduce computer volume and energy consumption. The introduction of layered two-dimensional materials has been reported to enhance the performance of the memristive electronic synapse. However, it is still a challenge to fabricate large-area layered two-dimensional films by scalable methods, which has greatly limited the industrial application potential of two-dimensional materials. In this work, a scalable pulsed laser deposition (PLD) method has been utilized to fabricate large-area layered SnSe films, which are used as the functional layers of the memristive electronic synapse with dual modes. Both long-term memristive behaviour with gradually changed resistance (Mode 1) and short-term memristive behavior with abruptly reduced resistance (Mode 2) have been achieved in this SnSe-based memristive electronic synapse. The switching between Mode 1 and Mode 2 can be realized by a series of voltage sweeping and programmed pulses. The formation and recovery of Sn vacancies were believed to induce the short-term memristive behaviour, and the joint action of Ag filament formation/rupture and Schottky barrier modulation can be the origin of long-term memristive behaviour. DFT calculations were performed to further illustrate how Ag atoms and Sn vacancies diffuse through the SnSe layer and form filaments. The successful emulation of synaptic functions by the layered chalcogenide memristor fabricated by the PLD method suggests the application potential in future neuromorphic computers.

## Abstract 2

Integration of short-term plasticity (STP) and long-term plasticity (LTP) in a single layered SnSe-based device has been achieved.

## Graphical abstract

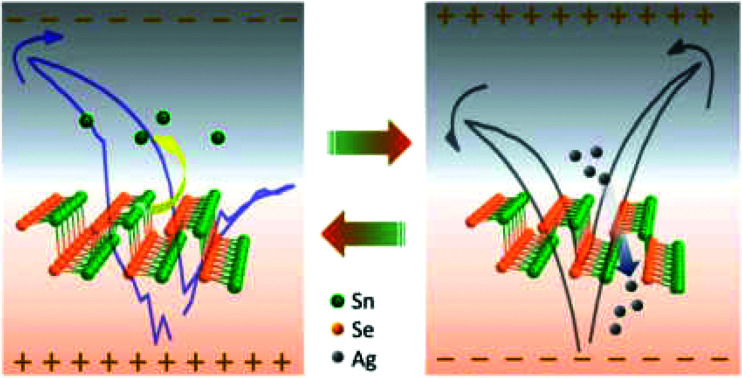

## Introduction

Neuromorphic computing has become a sparking research interest since great advances have been made in the artificial intelligence (AI) technology.^1,2^ Inspired by the human brain, which possesses the merits of large capacity in a small volume and low energy consumption, the utilization of an artificial synapse for neuromorphic computing is promising for next-generation AI research.^3–5^ Due to the history-dependent property, which is analogous to a synapse, the memristor is considered as a promising candidate unit that works as an electronic synapse.^5–7^ In addition, the high scalability and low operation power demands for memristors further enhance the advantage in electronic synapse applications. Several important synaptic functions, such as short-term potentiation (STP), long-term potentiation (LTP), spiking-rate-dependent plasticity (SRDP), and spike-timing-dependent plasticity (STDP), have been emulated by memristors.^5,8–14^ However, those that mimic both LTP and STP in the same memristor device have rarely been reported.^9,14–16^ For the learning and computing processes of neuromorphic computers, both LTP and STP are important as LTP is desirable for the memory function, while STP is indispensable for complex and temporal data analyses, which may highly improve the efficiency and reduce the resource occupation by less important data.^17–20^ Therefore, an electronic synapse possessing both LTP and STP properties should be able to offer the advantages of device scaling and circuit configuration simplification.

Although various materials have been employed in a memristive artificial synapse,^9,21–26^ the utilization of innovative two-dimensional (2D) materials has attracted increasing interest recently.^12,15,27–29^ Due to their special geometric structure, weak interlayer interaction, and special electronic and optical properties, 2D materials have revealed potential in both transparent and flexible memristor devices and memristors with enhanced performance. For example, by inserting atomic layer graphene in the ITO/ZnO/ITO structure, enhanced reliability was obtained with the suppressed variation of high-resistance states.^7^ The printable Ag/MoS_2_–MoO_*x*_/Ag structure exhibited an on/off ratio >10^6^, low operating voltage <0.2 V, and good bending fatigue resistance. However, the fabrication method of 2D materials is still a big challenge. Mechanical exfoliation and chemical vapor deposition (CVD) have been mainly employed to fabricate 2D material-based memristor devices. Low efficiency, poor uniformity, and scalability of the exfoliation method is obviously not compatible with industrial applications. Although large-area 2D materials can be fabricated by CVD method, the high temperature (>1000 °C) process is not industry-compatible. Thus, the exploration of the fabrication method of 2D materials, which is scalable and industry-compatible, is still highly desirable.

Recently, SnSe layered 2D structure came to our sight as a memristor candidate since it has been investigated as a supercapacitor, photo detector, and in thermoelectric devices.^30–34^ Stoichiometric SnSe has a high resistance and low-density of intrinsic defects.^35^ These properties make SnSe memory device multifunctional by integration of light sensitivity and ability to harvest energy from heat (SnSe possesses a narrow band gap and good thermoelectric property). In this work, a layered SnSe film made by scalable pulsed laser deposition (PLD) method was employed as the functional layer of the memristor. Both long-term and short-term memristive behaviours were obtained in the Ag/SnSe/NSTO device. The emulation of short-term potentiation, long-term potentiation and depression, SRDP and STDP as a synaptic emulator has been achieved in this SnSe-based memristor. The underlying mechanism for the dual mode memristor was illustrated with DFT calculation.

## Results and discussion

**Fig. 1:**
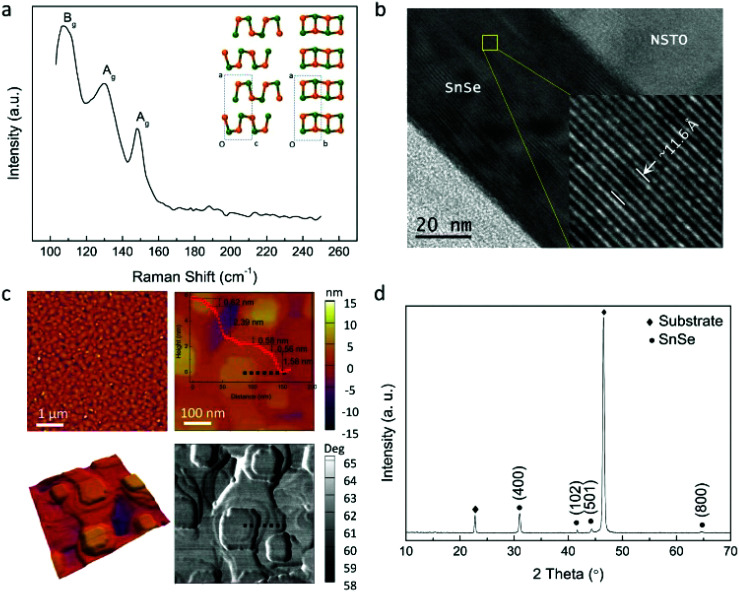
(a) Raman spectrum of the SnSe film on the NSTO substrate. The insets show the SnSe crystal structure along the *b* and *c* axes. (b) TEM image of the cross-section of the SnSe/NSTO structure. The inset displays high-resolution TEM image of the highlighted area with a yellow square. (c) Topography of the SnSe film on the NSTO structure. The images on the top are the AFM pictures in 5 and 2 μm area, respectively. The inset curve shows the step profile along the black dot line in the AFM image. The images on the bottom are the 3D profile and the corresponding phase image of the 2 μm area, respectively. (d) XRD results of the SnSe/NSTO structure.

The structure of the SnSe film was studied by means of Raman spectroscopy, transmission electron microscopy (TEM), scanning electron microscopy (SEM), atomic force microscopy (AFM), and X-ray diffraction (XRD). The Raman spectrum of the SnSe film is shown in Fig. 1a, where the peaks at 109, 130, and 150 cm^−1^ correspond to the B_g_ and two A_g_ modes of SnSe, respectively.^36^ The cross-sectional images of the SnSe/NSTO structure observed by TEM and SEM are shown in Fig. 1b and S1.† The flat surface and clear lattice fringes suggest very good quality of the SnSe film, where the lattice spacing of 11.6 Å is the *d*_(100)_ of SnSe. These results indicate that the SnSe film is epitaxially deposited on the NSTO substrate along the *a*-axis. The morphology of the SnSe film observed by AFM (Fig. 1c) exhibits square sheet-like islands stacked layer by layer, indicating the layer-island growth mode. The typical height of the steps measured from the AFM image of about 0.59 ± 0.03 nm corresponds to the interlayer spacing along the *a*-axis (2/*a*); this further proves the *a*-axis epitaxial growth of SnSe film. Fig. 1d shows the XRD pattern of the SnSe film, where a strong SnSe (400) peak can be identified, suggesting good crystallinity with a preferred [100] growth orientation of the film. Therefore, we conclude that the SnSe film fabricated by the PLD method can be regarded as a high-quality layered 2D material.

**Fig. 2:**
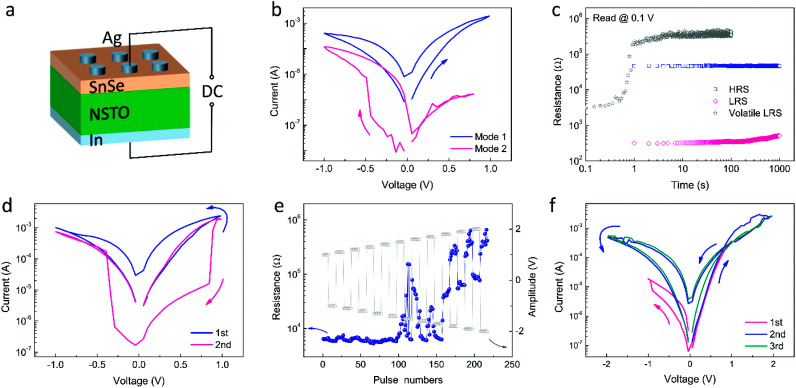
(a) Schematic diagram of the device structure and experimental configuration. (b) Typical *I*–*V* curves indicating two kinds of resistive switching behaviours. From the pink curve, when the negative voltage scan was applied first, the current suddenly jumps to 10^−5^ A during the negative voltage sweeping and returns to 10^−7^ A after the negative voltage is resumed to 0 V. On the contrary, according to the blue curve, when the voltage sweep starts from the positive side, the current gradually increases with increased positive voltage and retains the high conductive state even when the voltage sweeps back to the negative side. (c) Retention properties of Modes 1 and 2. The blue and pink dots show the HRS and LRS evolution after the set and reset process of Mode 1, while the gray dots exhibit the LRS evolution after the set process of Mode 2. (d) The *I*–*V* process of transition from Mode 1 to Mode 2. The first *I*–*V* curve in blue shows the *I*–*V* behaviour of Mode 1. Then, the second cycle in pink displays the change to Mode 2. (e) Transition between Mode 1 and Mode 2 induced by pulse series. When the positive and negative pulse series from ±1 V were applied, obvious resistance changes appear until the pulse amplitude is increased to 1.5 V. The resistance increases under the positive pulse series and decreases under the negative pulse series before −1.8 V pulses are applied. With increasing pulse amplitude from 1.8 V, the resistance further increases and then decreases under positive pulses. (f) The *I*–*V* process of transition from Mode 2 to Mode 1. The first pink curve shows the original short-term memory behaviour. The second blue curve shows the transition to long-term memory behaviour. The third curve shows the later long-term memory behaviour. The arrows indicate the direction of voltage sweep.

Electrical measurement was carried out on the SnSe/NSTO structure as shown in Fig. 2a and the typical *I*–*V* curves are shown in Fig. 2b. The dual mode resistive switching behaviours with both gradually changed resistance (Mode 1) and abruptly reduced resistance (Mode 2) can be obtained in the device. The two modes can be switched by altering the *I*–*V* scan directions (from positive to negative or from negative to positive). It is worth noting that Mode 1 often needs an activation operation such as a forming process with a forming voltage in the range of 1.0 to 2.5 V, which the volatile behaviour does not need. The memory properties of both resistive switching behaviours were investigated by resistance retention measurement, as shown in Fig. 2c. It is apparent that Mode 1 is non-volatile and shows good retention of both the high resistive state (HRS) and low resistive state (LRS) in the time range of 1000 s. The LRS of Mode 2 is volatile. It can only be retained for 1 s and then is gradually switched to HRS. The non-volatile and volatile resistive switching behaviours can be regarded as long-term and short-term memory, respectively.

The memristive behaviour of Mode 1 and Mode 2 can be transformed to each other through voltage sweep and pulse operation, as shown in Fig. 2d, where one can see that Mode 1 changes to Mode 2 due to a sudden reset process during positive sweeping. In the second cycle, the resistance jumps from the previous high resistive state of the first cycle (HRS_1_) to a higher resistive state HRS_2_ during positive voltage sweeping, and then drops to a relatively lower and volatile resistive state (LRS_2_) during negative voltage sweeping, indicating the transition from Mode 1 in the first cycle to Mode 2. A series of pulses can also realize the transition as shown in Fig. 2e. Beginning at LRS, the resistance changes through two processes when applying the pulse series with alternate polar and increased amplitudes. Under pulses with amplitudes within 1.5 to 1.8 V, the resistance increases under positive pulses and decreases under negative pulses; this indicates a negative setting process corresponding to Mode 2. The device switched to Mode 1 when the pulse amplitude is further increased.

The transition from Mode 2 to Mode 1 can also be achieved by voltage sweeping, as shown in Fig. 2f. As mentioned above, Mode 1 needs an activation process with larger voltage. As a result, increasing the maximum positive sweeping voltage and compliance current may realize the transition from Mode 2 to Mode 1. However, a training process may be needed to stabilize the memory behaviour after the transition process.

**Fig. 3:**
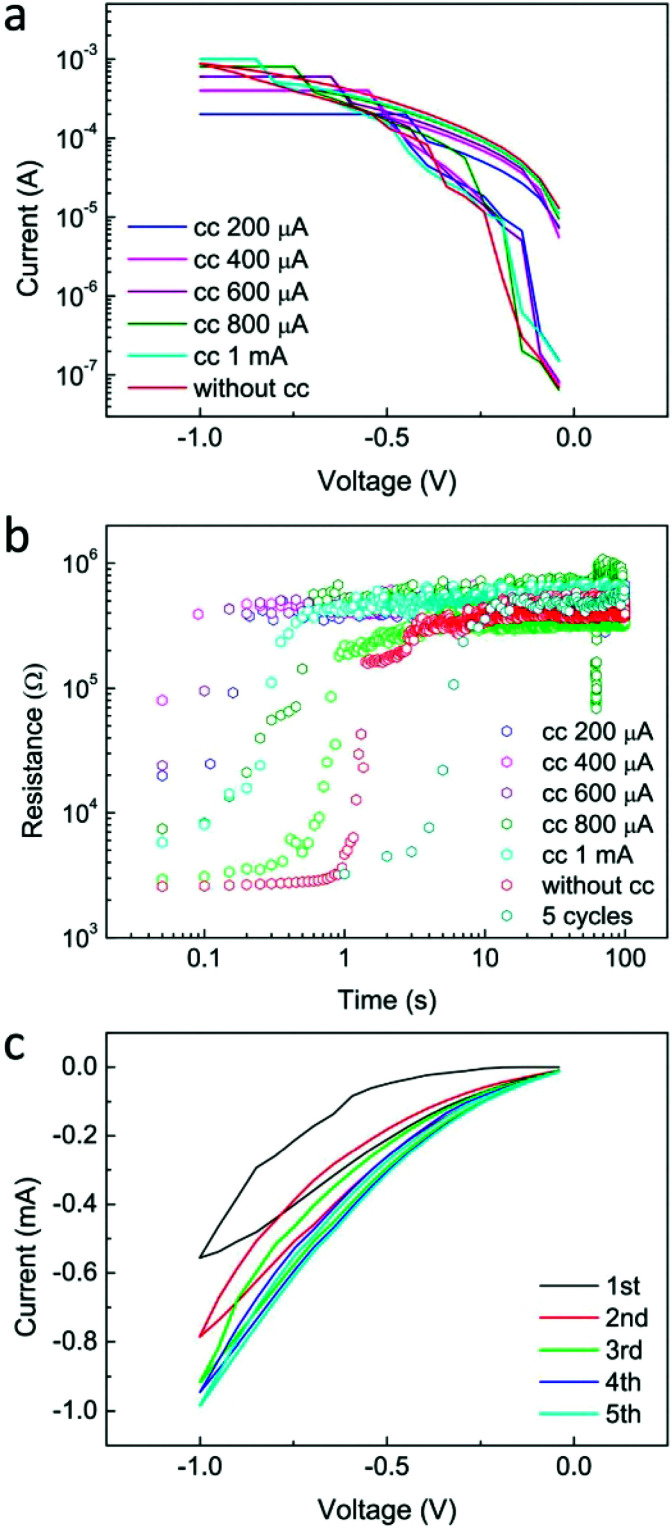
(a) *I*–*V* curves of the set process for short-term memory with varying compliance current. Increasing the low resistive state can be obtained while the compliance current increases. (b) Resistance retention properties after the set processes with different compliance current and cycle numbers. When increasing the compliance current from 200 μA to 1 mA or even without the compliance current, the retention time of the volatile LRS increases from less than 0.1 s to 1 s. Increasing the cycle number can greatly increase the retention time from 1 s to 10 s. The differences between the original resistances of the retention data must be attributed to the degradation during the delay time between the set process and retention measurement. (c) The *I*–*V* sweeps from 0 to −1 V 5 times. Increase in the current can be observed on increasing the cycle number.

The volatile memory property and plasticity operation of Mode 2 behaviour were further investigated. As shown in Fig. 3a, a series of set processes were performed with increasing compliance currents and it is obvious that the volatile LRS (resistance read from the back-sweeping *I*–*V* curves) reduces when the compliance current increases. The resistance retention behaviour after these set processes is displayed in Fig. 3b. One can see that the retention time increases when the compliance current increases. Subsequently, five cycles of *I*–*V* sweeping with different time intervals were performed. As shown in Fig. 3c, with a very short interval time (several milliseconds, which is confined by the instrument), the original HRS gradually decreases with cycle number, while it does not show any change with a longer interval time of several seconds. The potentiation and relaxation behaviours depending on interval time obviously indicate the presence of STP in the Ag/SnSe/NSTO/In device. The cycle-to-cycle and device-to-device variability of the short-term memory behaviour of the SnSe-based memristor is shown in Fig. S2.† It can be found that some of the devices repeat the *IV* curves well with the cycles, while the others show a variation in the set voltages. The device-to-device variability is not very good with varying high resistive state and set voltages, suggesting that the film uniformity needs to be further improved.

It is worth noting that the pulse width during the five cycles is much less than the former operations; this causes the obvious current decrease in the first cycle compared to that in Fig. 3a. Compared to the retention property of the resistance after 1 cycle and 5 cycles as shown in Fig. 3b, more cycles with longer sweeping time greatly increase the retention time from 1 s to 10 s. This dependence of the relaxation process on time and amount of charges (related to the compliance current) is usually due to the conducting nanofilament disruption and therefore, suggests a filament induced resistive switching mechanism.^15^

**Fig. 4:**
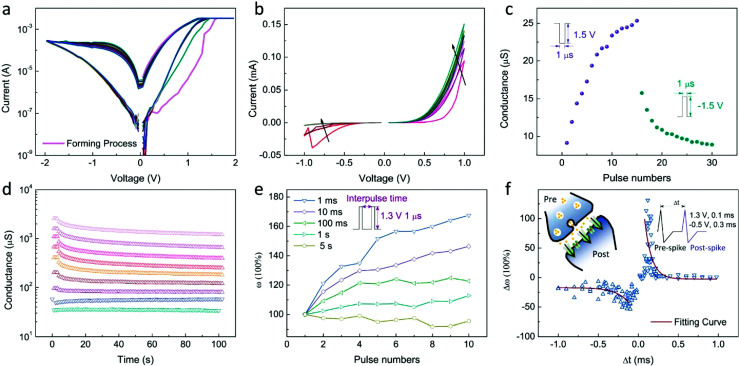
(a) Typical *I*–*V* curves of long-term memory behaviour for 50 cycles with a forming process shown in pink curve. (b) *I*–*V* sweep cycles from 0 to 1 V for 6 cycles and then 0 to −1 V for 4 cycles. (c) Conductance evolution with increasing pulse number. (d) Conductance varying with time in 100 s. (e) Synaptic weight changes with increasing pulse numbers and interval time. The inset shows the pulses applied for the measurement. (f) Synaptic weight changes with relative timing of the pre-spike and post-spike. The solid lines show the exponential fitting results. The left inset is the schematic of a biological synapse, while the right one shows the shapes of pre-spike and post-spike, which is applied on the Ag and NSTO electrode, respectively.

Investigations on Mode 1 of the device was also performed. Fig. 4a shows the typical *I*–*V* curves with a forming process, where stable and repeatable switching can be obtained in 50 cycles. The device-to-device variability of long-term memory behaviour the SnSe-based devices was studied in more than 5 cells with more than 5 cycles per cell, as shown in Fig. S3.† All the devices displayed good cycle-to-cycle repeatability. However, both HRS and LRS varied by more than one magnitude, which indicates a rather large device-to-device variability. The retention and endurance property at room temperature are also measured and are shown in Fig. S4.† The resistance of HRS varies slightly while that of LRS gradually degrades with time. It is worth noting that the LRS is read after the set process without the compliance current and the read voltage is 0.5 V. No degradation was observed in the endurance test within >10^5^ switching pulse cycles (±2 V, 1 μs).

By repeating the process of set and reset, as shown in Fig. 4b, one can see that the current increases gradually during the positive voltage sweeps, while it gradually reduces during the negative voltage sweeps. This memristive behaviour can be attributed to the growth and dissolution of silver islands^37,38^ and gradually changed the oxygen vacancies' concentration at the interface; this mechanism will be discussed later.

Fig. 4c shows the conductance modulation, which can represent the change in the weight in synaptic simulations, of the memory cell in the Ag/SnSe/NSTO/In device by pulse series. It is apparent that the conductance is strengthened by positive pulses and weakened by negative pulses, representing the potentiation and depression of the synaptic weight.^39^ Besides the sweep cycle and pulse number, the conductance or resistance of the device can be tuned by the compliance current, reset voltage, pulse amplitude, and width as well (shown in Fig. S5†), so the multilevel conductance can be easily obtained. Fig. 4d displays the retention property of the multiple conductance in 100 s. The degradation behaviour of the conductance can be attributed to the ion dynamics induced filament dissolution.

Synaptic plasticity, which represents the strength of the connection between two neuron cells, is an important function in the learning and memory process. It was emulated in the device, as shown in Fig. 4e, where the spiking rate influence on the synaptic weight can be described as SRDP.^40^ One can see that by applying a pulse of 1.3 V with pulse-to-pulse intervals from 1 ms to 5 s, the conductance of the memristor increases in a varying rate. For the sequence of interval shorter than 10 ms, the conductance increases in a similar slope, while on further increasing the intervals, the conductance increases more slowly and eventually saturates at the interval of 5 s. This result successfully demonstrates the SRDP behaviour, *i.e.*, the weight enhancement is influenced by the rate of applied pulses.

On the other hand, the STDP rule works by adjusting the synaptic plasticity through relative timing of a neuron's input and output spikes.^41^ For a synapse, if the pre-spike precedes the post-spike, the connection between the neurons becomes stronger and long-term potentiation occurs.^42^ If the post-spike precedes the pre-spike, the connection becomes weaker and long-term depression occurs. In Fig. 4f, Δ*t* is defined as the interval time between pre- and post-synaptic spikes with the amplitude of 1.3 V and width of 100 μs, and the change in the synaptic weight Δ*ω* is defined as (*G*_2_ − *G*_1_)/*G*_1_, where *G*_1_ and *G*_2_ are the conductance obtained before and after the pulse pairs are removed, respectively. It was found that irrespective of positive or negative Δ*t*, the conductance shows negative correlation with Δ*t*, *i.e.*, when Δ*t* increases, the absolute value of Δ*ω* decreases. Therefore, STDP property is successfully mimicked in the Ag/SnSe/NSTO structure.

**Fig. 5:**
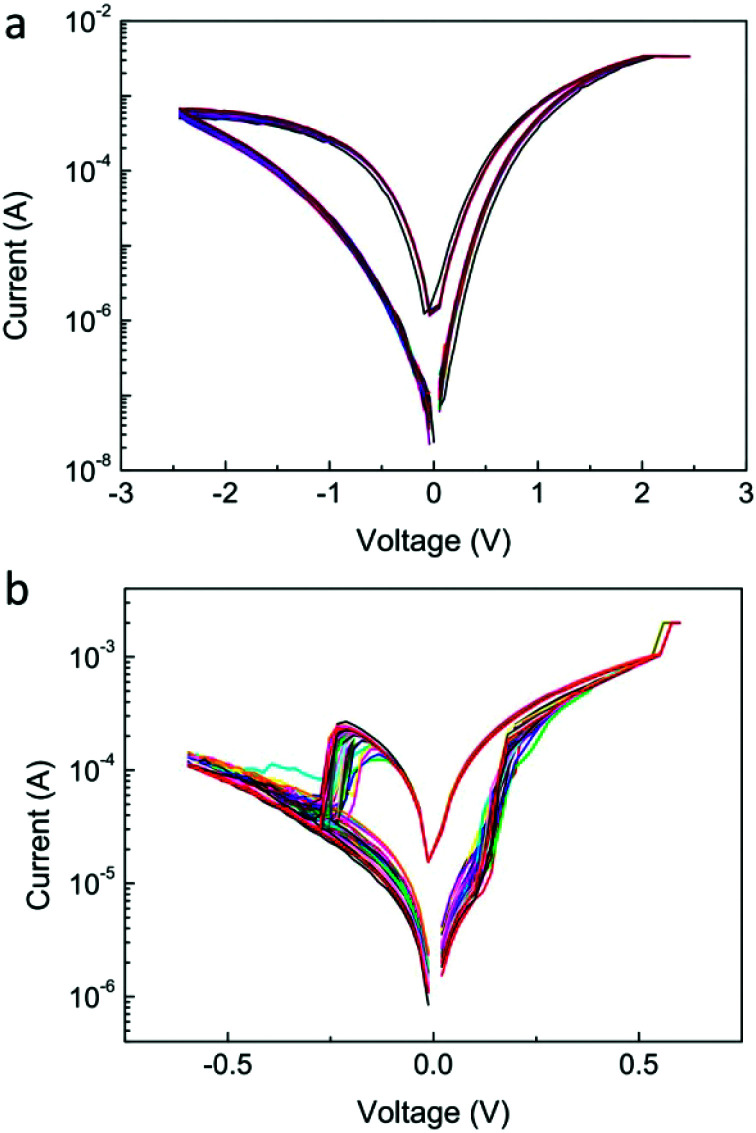
*I*–*V* curves of 50 cycles in (a) Au/SnSe/NSTO/In and (b) Ag/SnSe/Pt devices.

In order to find out the underlying mechanism responsible for the memristive behaviour, the device structures of Au/SnSe/NSTO/In and Ag/SnSe/Pt were studied for comparison, and the results are shown in Fig. 5. As neither active metal electrode nor oxide functional material exists in the Au/SnSe/NSTO/In device, the switching behaviour could be induced by variation in the Schottky barrier caused by the oxygen vacancies' redistribution at the SnSe/NSTO interface.^43^ As shown in Fig. 5a, the Au/SnSe/NSTO/In device exhibits similar *I*–*V* curves with the Ag/SnSe/NSTO/In device. However, the on/off ratio of the Ag/SnSe/NSTO structure is larger than that of the Au/SnSe/NSTO structure. The different *R*_HRS_ could be attributed to the work function difference of silver and gold electrodes, which results in higher Schottky barrier at the SnSe/NSTO interface. On the other hand, as shown in Fig. 5b, the *I*–*V* curve of the Ag/SnSe/Pt device shows abrupt set and reset processes. The bipolar resistive switching of the Ag/SnSe/Pt device matches well with the mechanism of metal redox induced filament formation and rupture. Due to the ultralow set and reset voltages, which are about 0.2 and −0.3 V, respectively, the Ag filament formation and rupture processes are inevitable in the resistive switching of the Ag/SnSe/NSTO/In device. The formation and rupture of the Ag filament in SnSe was observed by TEM as shown in Fig. S6.† Moreover, both the resistances at HRS and LRS of the Ag/SnSe/Pt device are lower than those of the Ag/SnSe/NSTO/In device and the resistance difference between the HRS of the two devices is larger than that between LRS, indicating the effect of coexistence of filament and interface in the Ag/SnSe/NSTO/In device, as illustrated in Fig. S7a and b.†

As a result, the resistive switching of the non-volatile e Mode 1 in the Ag/SnSe/NSTO/In device is induced by the joint action of Ag filament formation/rupture and interface effect involving the redistribution of oxygen vacancies. Furthermore, since the volatile Mode 2 is activated by negative electric field at a relatively high resistance state, the Ag from the top electrode and O^2−^ from NSTO involved in the redox process can be excluded. The creation and recovery of Sn vacancies may be responsible for the volatile behaviour, as illustrated in Fig. S7c.† Due to the fast recovery of Sn vacancies, which results in short retention time, the transition from volatile Mode 2 to non-volatile Mode 1 often needs merely a forming process. However, slight structural destruction is inevitable due to fast switching and thermal effect. Mode 1 switched from Mode 2 may be unstable and needs a training process to be stabilized, as mentioned before. On the other hand, the transition from Mode 1 to Mode 2 is relatively harder since a large amount of silver ions are injected into the SnSe film to form the filament, making the structural destruction more serious. Therefore, more voltage sweeping cycles and pulse series are needed to further destroy the Ag filament and increase the resistance of the SnSe film.

**Fig. 6:**
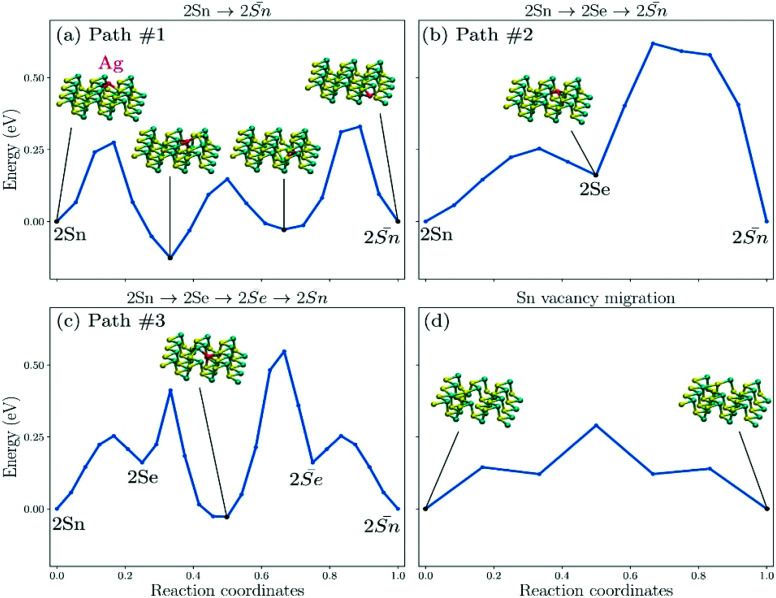
(a–c) The minimum energy path and activation energy for an Ag atom crossing SnSe layer *via* 3 paths. (d) The minimum energy path of Sn-vacancy migration in the SnSe layer.

To further illustrate the formation of the filament across the SnSe layers, density functional theory (DFT) calculations were performed to evaluate the possibility of Sn-vacancy and Ag migrations in SnSe. Because of the large number of atoms when considering multiple layers of SnSe together with the computationally intensive nudged elastic band (NEB) calculations, which considers multiple images of the structure, we studied migrations on (and across) a single layer of SnSe. We do not expect the presence of additional layers to significantly change the activation barriers computed on a single layer. We identified two intercalated sites between the layers of bulk SnSe corresponding to two equivalent adsorbed sites on the monolayer. We validated that the low energy intercalated site of Ag in bulk SnSe, corresponding to Ag making 3 bonds with Sn and 1 bond with Se in a tetragonal manner, is also the low energy adsorbed (and equivalent) site on the monolayer, corresponding to a pyramidal geometry for which the Sn bond to the upper layer is missing. The two possible adsorption sites on monolayer SnSe are labelled by the number and nature of the dominant bond with Ag and are represented in Fig. 6. Therefore, the ground state adsorption site discussed above represents a 2Sn site and the second lowest energy site represents a 2Se site.

We computed the energy required for a silver atom to cross the layer of SnSe. If we consider the initial and final adsorption sites of Ag to be its ground state 2Sn, cross-layer diffusion can be achieved *via* 3 different paths. Path #1 represents the direct migration of Ag between two 2Sn sites, on each side of the SnSe layer (Fig. 6a). We note that the corresponding potential energy surface presents two minima, when the Ag atom is located inside the layer of SnSe. This type of interstitial site, inside the monolayer, has been observed previously in 2D materials.^44^ The activation energy of the barrier to overcome so as to achieve cross-layer diffusion represent 0.27, 0.27, and 0.36 eV. Path #2 is two-folds and corresponds to Ag first migrating from the low energy 2Sn site to 2Se, on the same side of the monolayer (0.25 eV), followed by the cross-layer diffusion from 2Se to 2Sn, on the other side of the monolayer (Fig. 6b). This last transition represents an activation barrier of 0.46 eV. Finally, path #3 is 3-folds, starting with identical 2Sn to 2Se transition as in path #2, followed by the cross-layer diffusion from 2Se to 2Se on the other side of the layer, and the transition from 2Se to 2Sn (Fig. 6c). The second step includes a local minimum inside the single layer of SnSe. The final step is the mirror PES of the first step. The activation energies of path #3 are 0.25, 0.25, 0.58, and 0.25 eV, respectively. We also computed the minimum energy path of Sn-vacancy migration and found the activation energy of 0.29 eV (Fig. 6d). The activation barriers are reported Table 1.

**Table 1:** Activation energy (in eV) corresponding to the barriers presented in Fig. 6

	*E* ^1^_A_	*E* ^2^_A_	*E* ^3^_A_	*E* ^4^_A_
Path #1	0.27	0.27	0.36	—
Path #2	0.25	0.46	—	—
Path #3	0.25	0.25	0.58	0.25
Sn-vac	0.29	—	—	—

These DFT calculations indicate that crossing a layer of SnSe is possible with an average energy barrier of 0.36 ± 0.12 eV, which is within the distribution of activation energy of common ReRAM based on Cu migration in amorphous dielectrics.^45^ Although path #1 includes multiple barriers to overcome, it represents the most probable cross-layer diffusion path because of the corresponding low activation energies. However, we found that Sn-vacancy migration energy is even lower than that for Ag cross-layer diffusion. Therefore, DFT calculations predict that the principal migration mechanism at low voltage is dominated by vacancy diffusion whereas Ag migration and cross-layer diffusion are indeed possible and energetically similar than those at the origin of switching in common ReRAM. These results suggest that dual mechanisms can exit in the same structure of Au/SnSe/NSTO/In.

## Conclusions

A high-quality layered SnSe film fabricated by scalable PLD method was utilized as a functional layer of memristors. Synaptic functions STP and LTP and transition of short-term and long-term memory have been obtained in the layered SnSe-based memristor device. Supported by DFT calculations, Sn vacancy migration and Ag filament formation across the layers of SnSe are believed to be responsible for the short-term and long-term behaviour, respectively. The scalable PLD method used in SnSe film fabrication indicates the industrial application potential of 2D materials. The controllable transition between the short-term and long-term memory behaviour may provide a path to design a multifunctional memory-storage compounded device for future energy-efficient neuromorphic computing or displaying application.

## Experimental

### Material preparation and device fabrication

The SnSe film with a thickness of ∼50 nm was deposited by pulsed laser deposition (PLD) method on a commercially available 5 mm × 5 mm Nb-doped STO(001) substrate (Nb concentration is 0.7%). The deposition temperature was 330 °C and the background pressure was 5 × 10^−6^ Torr. A KrF excimer laser was used as the laser source, with the energy of 270 mJ and repetition of 1 Hz. The distance between the substrate and the SnSe target was 30 mm. After that, the silver electrode was deposited on the film with a magnetron sputtering system. The deposition was performed at room temperature under the Ar pressure of 4.7 mTorr. With the help of a metal mask, the silver electrode with a diameter of 100 μm and thickness of ∼100 nm was obtained. In order to facilitate the electric measurement, the In bottom electrode was pressed on the bottom of the NSTO substrate, thus constructing ohmic contact at the NSTO/In interface.

### Material characterization and electronic property measurement

The structure of the SnSe/NSTO device was measured with X-ray diffraction (XRD) (Rigaku SmartLab) and transmission electron microscopy (TEM) (JEOL 2100 F). The morphology and current map of the films was obtained with the atomic force microscope (Asylum Research MFP 3D Infinity). The electrical performance and synaptic plasticity emulation were performed at room temperature with an Arc One measurement instrument. Electric field was applied on the Ag electrode with the NSTO electrode grounded.

### DFT calculation

DFT calculations were performed with VASP^27,46^ within the generalized gradient approximation proposed by Perdew, Burke, and Ernzerhof.^47^ We used Grimme's DFT-D3 method^48^ to correct for the van der Waals interaction poorly described by standard DFT. For all the DFT calculations, we used a kinetic energy cutoff of 500 eV and a *k*-mesh of 2 × 2 × 1. Convergence was achieved when energy and forces reached a minimum value of 1 × 10^−7^ eV and 1 × 10^−6^ eV, respectively. For NEB calculation, we use looser convergence criteria of 1 × 10^−5^ eV and 5 × 10^−2^ eV Å^−1^ for energy and force, respectively. All the calculations include spin polarization.

## Conflicts of interest

There are no conflicts to declare.

## Supplementary Material

NA-002-C9NA00447E-s001
