# Supplementary material for: A dual mode electronic synapse based on layered SnSe films fabricated by pulsed laser deposition
Source: Nanoscale Adv. 2020 Jan 20;2(3):1152–60. doi: 10.1039/c9na00447e (PMC9418994; doi:10.1039/c9na00447e)
Supplement: NA-002-C9NA00447E-s001 [file NA-002-C9NA00447E-s001.pdf]

Electronic Supplementary Material (ESI) for Nanoscale Advances.
This journal is © The Royal Society of Chemistry 2020

Supplementary material

Dual Modes Electronic Synapse Based on Layered SnSe Films Fabricated by Pulsed Laser Deposition

Xinxin Chen[a], Chun-Hung Suen[a], Hei-Man Yau[a], Feichi Zhou[a], Yang Chai[a], Xiaodan Tang[b], Xiaoyuan Zhou[b,*], Nicolas Onofrio[a,*] and Ji-Yan Dai [a,*]

[a] *Department of Applied Physics, The Hong Kong Polytechnic University, Hung Hom, Kowloon, Hong Kong*

[b] *College of Physics, Chongqing University, Chongqing 401331, P. R. China.*

** Corresponding Author*

E-mail addresses: jiyan.dai@polyu.edu.hk (J.-Y. Dai), nicolas.onofrio@polyu.edu.hk (N. Onofrio) and xiaoyuan2013@cqu.edu.cn (X. Zhou).

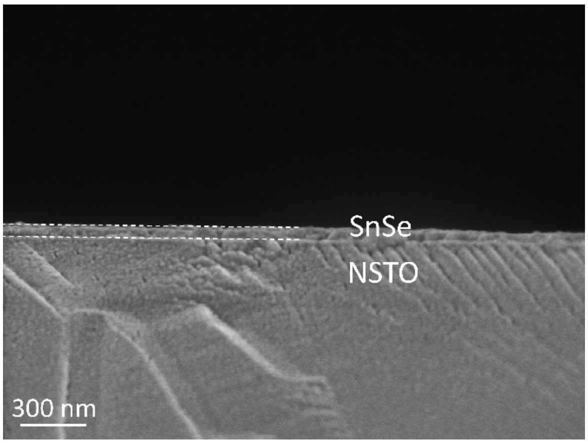

Fig. S1 SEM cross-section image of SnSe/NSTO structure

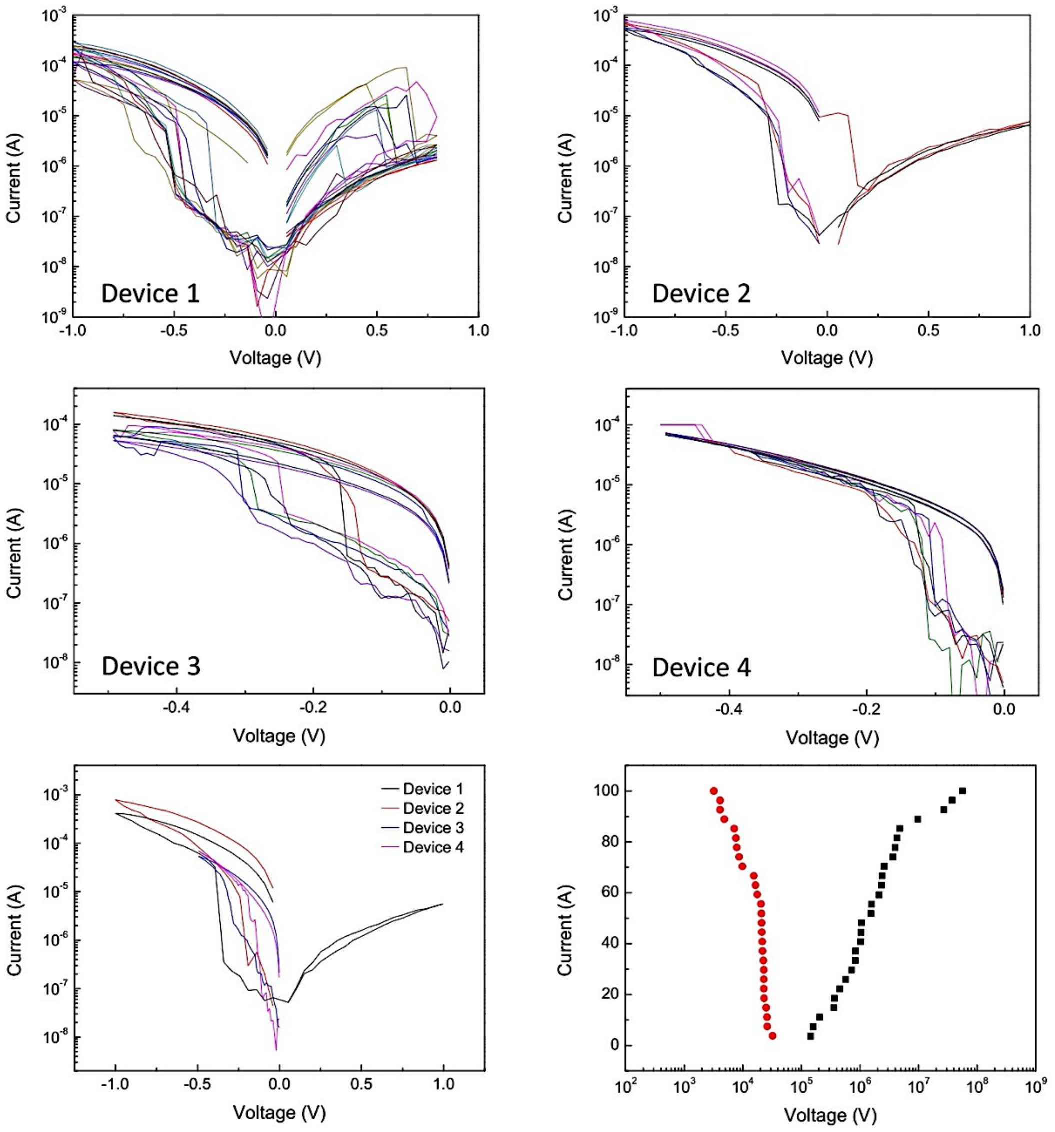

Fig. S2 Cycle-to-cycle and device-to-device variability of the SnSe-based memory device.

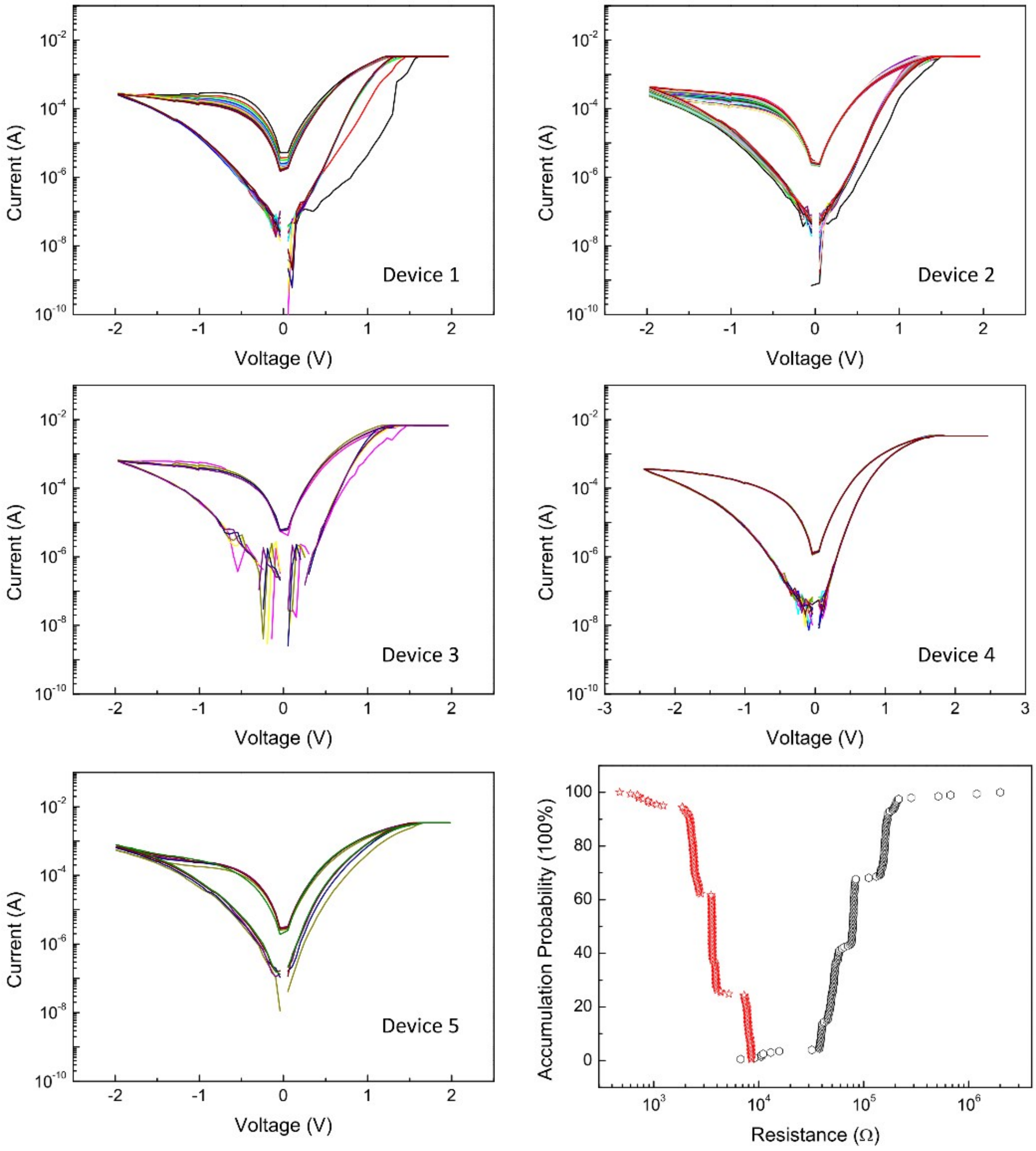

Fig. S3 Cycle-to-cycle and device-to-device variability of the SnSe-based memory device.

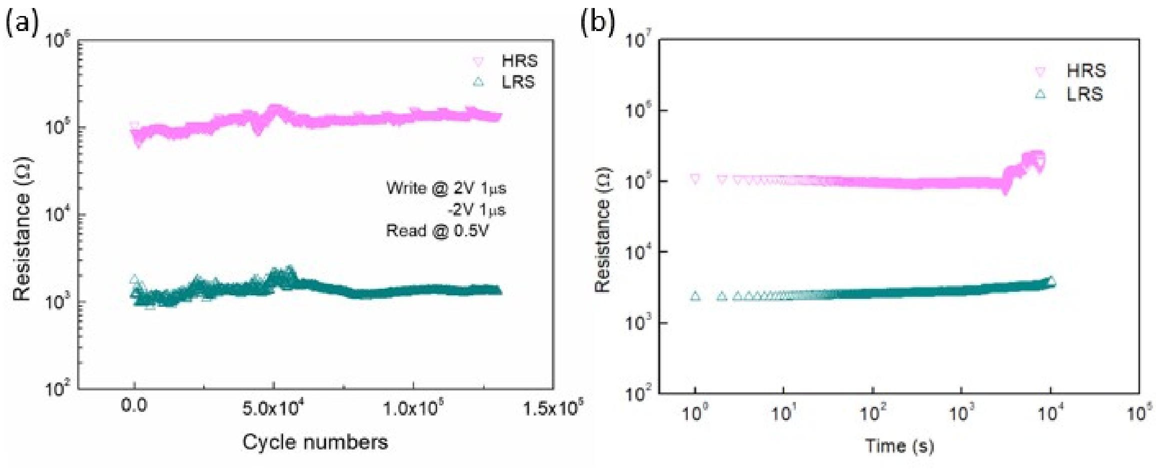

Fig. S4 Endurance and retention of the SnSe-based memory device.

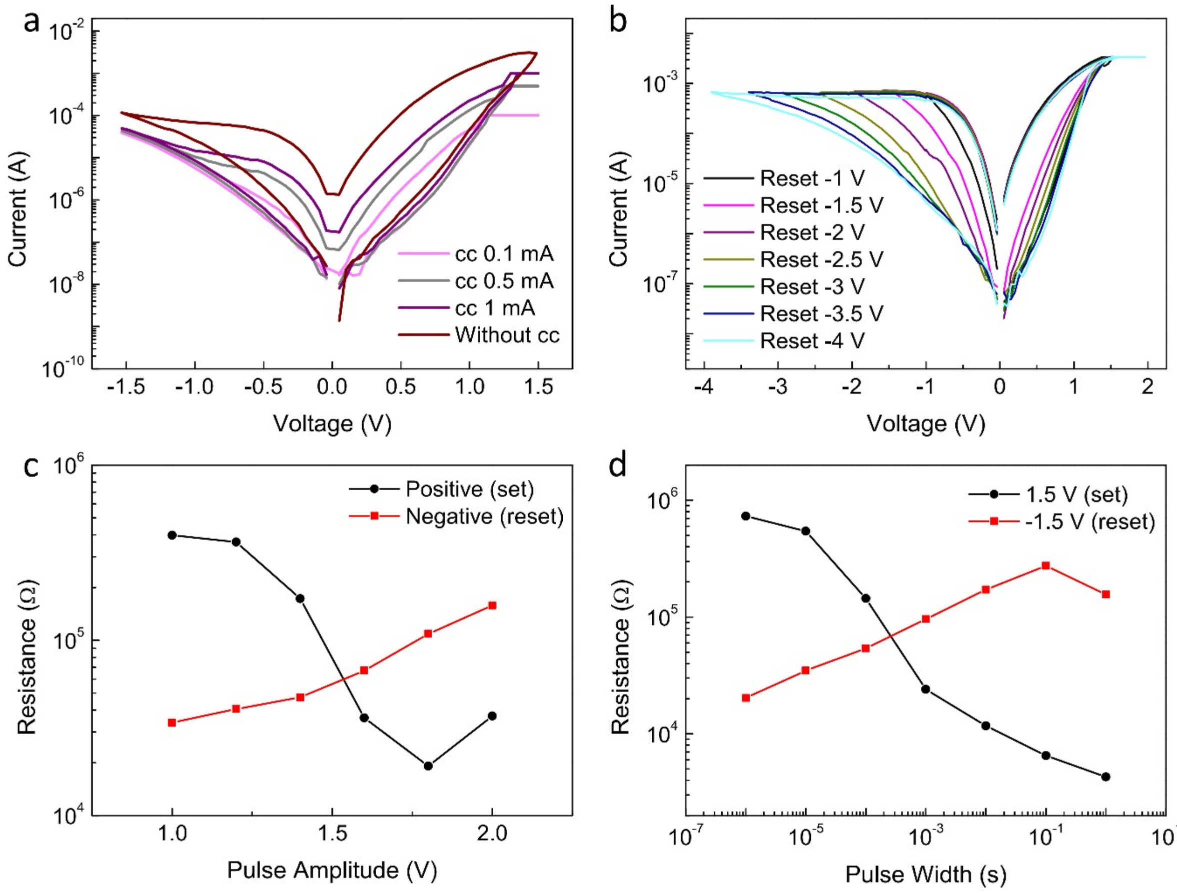

Fig. S5 (a) I-V curves with increasing compliance current. (b) I-V curves with increasing sweep voltage range. (c) Resistance dependence on pulse amplitude. (d) Resistance dependence on pulse width.

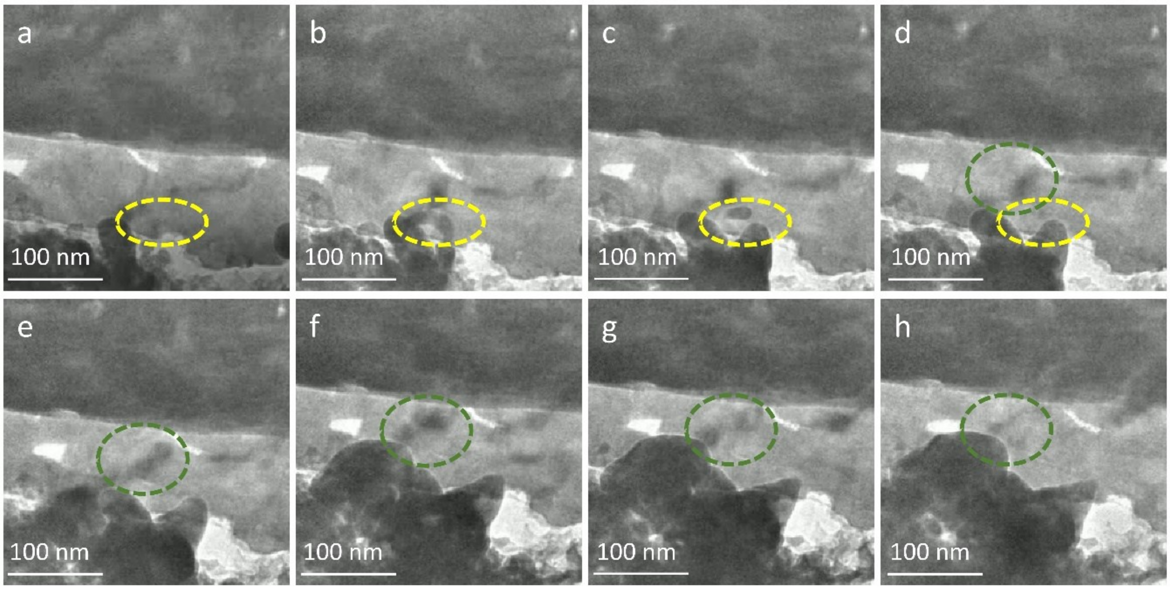

Fig. S6 TEM images displaying the formation and dissolution of Ag filament in SnSe film under electric field.

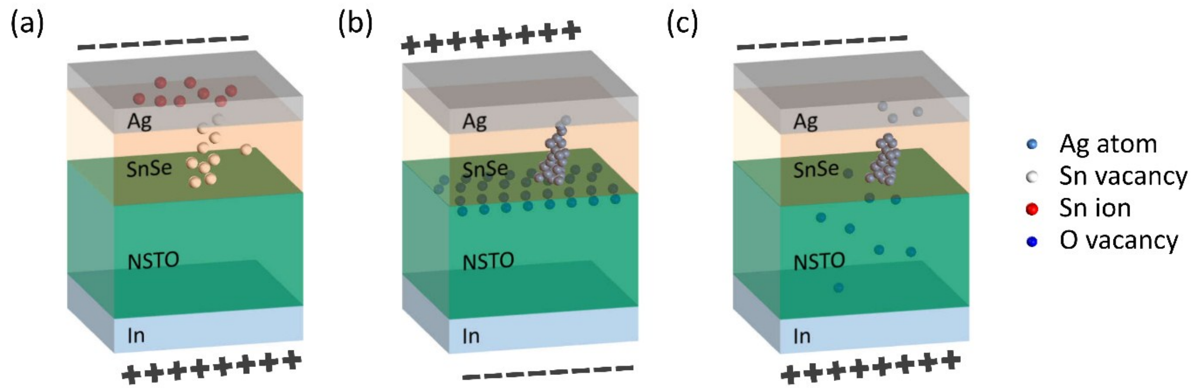

Fig. S7 (a) Mechanism for LRS of nonvolatile resistive switching. (b) Mechanism for HRS of nonvolatile resistive switching. (c) Mechanism for volatile LRS.
